# Mediation effects of subjective social status and personality traits between childhood nurturing experiences and depressive symptoms in adult volunteers

**DOI:** 10.1002/pcn5.70031

**Published:** 2024-11-03

**Authors:** Yuki Nakagawa, Miki Ono, Chihiro Morishita, Mina Honyashiki, Yu Tamada, Yota Fujimura, Shinji Higashi, Naoki Hashimoto, Takeshi Inoue, Jiro Masuya

**Affiliations:** ^1^ Department of Psychiatry Tokyo Medical University Tokyo Japan; ^2^ Department of Psychiatry Tokyo Medical University Hachioji Medical Center Tokyo Japan; ^3^ Department of Psychiatry Tokyo Medical University Ibaraki Medical Center Ibaraki Japan; ^4^ Department of Psychiatry Hokkaido University Hokkaido Japan

**Keywords:** depressive symptoms, five‐factor model of personality, nurturing experience in childhood, structural equation modeling, subjective social status

## Abstract

**Aim:**

Various risk factors, such as childhood nurturing experiences and subjective social status, have been identified to be involved in the onset of depression. However, the mechanism of depression is not yet fully understood. In this study, we hypothesized that nurturing experienced in childhood affects subjective social status and current personality traits, which in turn influence depressive symptoms in adulthood, and verified this hypothesis through structural equation modeling.

**Methods:**

A questionnaire survey was conducted on 404 adults. Multiple regression analysis and structural equation modeling were conducted using demographic information and scores for the Patient Health Questionnaire‐9, Parental Bonding Instrument, and NEO Five‐Factor Inventory.

**Results:**

Subjective social status was found to mediate the association between nurturing experiences and neuroticism (0.029 for Overprotection and –0.034 for Care). On the other hand, neuroticism was found to mediate the association between subjective social status and depressive symptoms (–0.097 in Care model and –0.103 in Overprotection model), as well as the association between nurturing experiences and depressive symptoms (0.144 for Overprotection and –0.134 for Care). Furthermore, it was also shown that complex paths, in which the association of nurturing experiences with depressive symptoms was mediated by two factors, namely, subjective social status and neuroticism, were statistically significant as indirect effects (0.016 for Overprotection and –0.018 for Care).

**Conclusion:**

In this study, we clarified that nurturing experienced in childhood affects neuroticism in adulthood, which is mediated by subjective social status, and furthermore, the effects of nurturing on neuroticism lead to varying levels of depressive symptoms in adulthood. The mediation effects demonstrated in the present study may contribute towards unraveling the causes of depression and developing effective treatments for depressive symptoms.

## Graphical abstract

Only neuroticism among the five NEO‐FFI personality traits mediated the influence of subjective social status (SSS) on depressive symptoms (PHQ‐9), whereas the other four traits did not. A significant indirect influence was observed, wherein childhood nurturing experiences (PBI) impacted neuroticism through SSS and further affected depressive symptoms in adulthood. PBI, Parental Bonding Instrument; PBI Care, PBI care factor; PBI OP, PBI overprotection factor; N, neuroticism; A, agreeableness; E, extraversion; C, conscientiousness; O, openness.

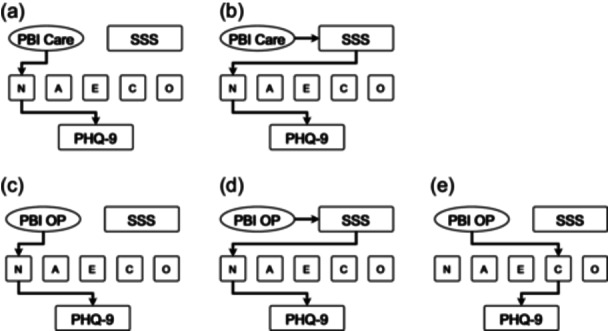

## INTRODUCTION

Depression is a common mental disorder with a lifetime prevalence in the Japanese population of 5.7%.^1^ Various factors, such as genetics, personality traits, and environmental influences, are reported to be risk factors for the onset of depression.^2^, ^3^, ^4^ Regarding personality factors, attributes such as neuroticism, low self‐esteem, and affective temperament have been demonstrated to influence depressive symptoms in adulthood.^5^, ^6^, ^7^ On the other hand, among the environmental factors, adverse childhood experiences, such as unfavorable parenting styles, have been shown to impact the onset of adulthood depression.^3^, ^8^, ^9^, ^10^

As there is a large time interval between nurturing experienced in childhood and adulthood depression, it is conceivable that certain mediating factors bridge this gap. Previous research has reported that dysfunctional family experiences influence depressive symptoms in adulthood, with neuroticism acting as a mediator.^5^, ^11^ Additionally, individual personality traits, such as self‐esteem, affective temperament, and resilience, reportedly act as mediating factors between nurturing experienced in childhood and depressive symptoms in adulthood.^5^, ^6^, ^12^, ^13^, ^14^ These findings suggest that the personality of individuals might be a pivotal mediating factor in the influence of childhood experiences on depression in adulthood.

In terms of classifying personality traits, the five‐factor model (FFM) stands out as a consensus method. The FFM describes personality through the following five general traits: extraversion, neuroticism, openness to experience, conscientiousness, and agreeableness.^15^, ^16^, ^17^ In 1989, Costa and McCrae developed the Revised NEO Personality Inventory (NEO‐PI‐R) to measure these five characteristics in healthy adults, and it is still widely used in research today.^18^, ^19^ Among these traits, high scores in neuroticism or low scores in extraversion are reportedly linked with the onset of depression.^20^, ^21^, ^22^ However, to the best of our knowledge, no studies to date have comprehensively investigated the associations among all five personality traits, depression, and nurturing experienced in childhood.

Recently, the association between subjective social status (SSS) and health has garnered attention. SSS measures individuals' perceptions of their place within the societal hierarchy.^23^ It has been shown to impact health as much as, if not more than, objective socioeconomic status, which includes income, occupation, and years of education.^24^, ^25^, ^26^, ^27^ SSS is associated with the prevalence of various psychiatric disorders, including mood disorders, anxiety disorders, and substance use disorders.^28^ In particular, reports have suggested a link between low SSS and the prevalence of depression.^28^, ^29^

Considering the emerging focus on SSS as a factor in the onset of depression, some studies have reported that SSS and self‐esteem mediate the effects of childhood experiences on depressive symptoms.^6^ Other research has highlighted the mediating role of psychosocial vulnerability, including neuroticism, in the impact of SSS on health.^30^

From these considerations, it is plausible that personality traits, such as self‐esteem and neuroticism, might act as significant mediating factors when SSS impacts health, including depression. However, to our knowledge, no study to date has comprehensively investigated the associations among all five personality traits of the FFM and SSS, as well as their impact on depressive symptoms. Instead of focusing on specific personality traits as mediating factors in the association of SSS with depressive symptoms, we believe that investigating this through a comprehensive FFM would eliminate methodological bias and elucidate the role of personality traits in adulthood depression.

From these previous findings, both SSS and personality traits can be considered to be crucial elements that mediate the effects of early childhood experiences on depressive symptoms in adulthood. According to Parker et al., parents' nurturing attitudes towards their children during early childhood can be categorized into two factors: care and overprotection.^31^ SSS tends to develop relatively stably from adolescence into adulthood.^32^ Moreover, the NEO‐PI‐R, which measures the five‐factor personality traits, was created specifically for adult subjects. Consequently, we hypothesized that the care and overprotection components of nurturing experienced in childhood influence SSS, which further impacts current adult personality traits, and subsequently affects depressive symptoms in adulthood. The purpose of this study was to verify this hypothesis through structural equation modeling in adult volunteers.

## METHODS

### Subjects

This study was performed from January to August 2014 as part of a larger study.^12^ Japanese adult community volunteers were recruited using the convenience sampling method. They were informed about the study through the authors' acquaintances and flyers that were distributed at a university in Japan. A self‐administered questionnaire was distributed to 853 adult volunteers from January to August 2014, and valid responses with written consent were returned by 404 individuals (220 men and 184 women; average age: 42.3 ± 11.9 years). The inclusion criterion was set as adults aged 20 years and older. Exclusion criteria were those with a severe physical illness or organic brain disease. An anonymous survey was conducted using the four psychological questionnaires listed below and a demographic information questionnaire. Subjects were informed that: (1) participation in the study was not mandatory and could be determined by free will; (2) there would be no disadvantage in not participating; and (3) data management would be coded in a nonidentifiable format to ensure that personal information would not be leaked externally. Those who provided consent in writing participated in the study. This study was conducted according to the Helsinki Declaration (revised in Fortaleza in 2013), and was implemented with the approval of the Medical Ethics Review Committee of Tokyo Medical University (SH3308) and Hokkaido University Hospital (013‐0184).

### Self‐administered questionnaires

#### Patient Health Questionnaire‐9

The Patient Health Questionnaire‐9 (PHQ‐9) is a self‐administered depression rating scale consisting of nine items.^33^ The Japanese version of the PHQ‐9 was developed by Muramatsu et al., and its reliability and validity have been verified.^34^ The total score for the PHQ‐9 (0–27) as the severity of depressive symptoms was used for the analysis. The Cronbach's α coefficient for the total score for the PHQ‐9 was 0.849, indicating high internal consistency.

#### Parental Bonding Instrument

The Parental Bonding Instrument (PBI) is a subjective rating scale used to evaluate the nurturing attitude received from parents during childhood.^31^ Parker et al. posited that the nurturing attitude of parents, as perceived by the child, is condensed into two factors, that is, “Care” and “Overprotection,” and the PBI consists of 25 items, with 12 under “Care” and 13 under “Overprotection.”^31^ A higher Care score indicates a higher tendency for parental care (lower tendency for indifference or rejection), and a higher Overprotection score indicates a higher tendency for parental overprotection (lower tendency to encourage independence). Long‐term stability of the PBI over a 20‐year period was reported.^35^ The Japanese version of the PBI has been developed by Kitamura and Suzuki, and its validity and reliability have been confirmed.^36^ The Cronbach's α coefficients for the individual subscales of the PBI were found to be 0.909 for paternal care, 0.854 for paternal overprotection, 0.917 for maternal care, and 0.870 for maternal overprotection, and indicate high internal consistency.

#### NEO Five‐Factor Inventory

The NEO Five‐Factor Inventory (NEO‐FFI) is a personality test that assesses the FFM of personality traits, which is closely associated with the Big Five personality traits. The NEO‐FFI is a shortened version of the 240‐item NEO‐PI‐R and was developed for more convenient implementation.^37^ The NEO‐FFI consists of 60 items, with 12 questions for each of the five factors (extraversion, neuroticism, openness to experience, conscientiousness, and agreeableness), and measures the five personality traits based on the scores for each question. The Japanese version of the NEO‐FFI was developed by Shimonaka et al.,^38^ and its reliability was confirmed.^19^ In this study, the scores for each of the five factors were used for analysis. The Cronbach's α coefficients for the individual subscales of the NEO‐FFI were found to be 0.818 for extraversion, 0.860 for neuroticism, 0.573 for openness to experience, 0.725 for conscientiousness, and 0.798 for agreeableness. These results were generally similar to previous studies on the reliability of the Japanese version of the NEO‐FFI in large Japanese community samples.^39^

#### Subjective social status

Subjective social status (SSS) measures people's perception of their position within the societal hierarchy. It is evaluated subjectively in 10 levels based on the question: “If society is divided into layers from 10 to 1 in order from the top, where do you think you belong?”^40^ SSS is associated with the prevalence of several mental illnesses, such as mood disorder, anxiety disorder, and substance use disorder, as revealed in large‐scale surveys.^28^

#### Statistical analysis

Statistical analyses were conducted using SPSS Statistics 28 software (IBM) and Mplus Version 8.5 software (Muthén & Muthén).

**Figure 1:**
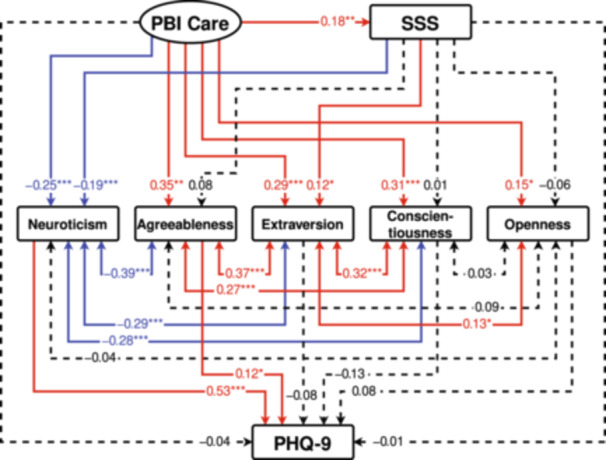
Results of covariance structure analysis in the structural equation model with the Care subscale on the Parental Bonding Instrument (PBI Care), the subjective social status (SSS) score, and scores of the five personality traits (neuroticism, agreeableness, extraversion, conscientiousness, and openness) of the NEO Five‐Factor Inventory, as well as depressive symptoms (Patient Health Questionnaire‐9 [PHQ‐9] score) in 404 adult volunteers. Rectangles indicate the observed variables. An oval indicates the latent variable, which consists of two observed variables, namely, paternal and maternal care subscores (not shown in this figure). Solid arrows indicate statistically significant pathways, red arrows indicate positive effects, and blue arrows indicate negative effects. Dashed lines indicate nonsignificant pathways. The numbers next to the arrows indicate the direct standardized path coefficients (minimum: −1; maximum: +1). *p < 0.05, **p < 0.01, and ***p < 0.001.

**Figure 2:**
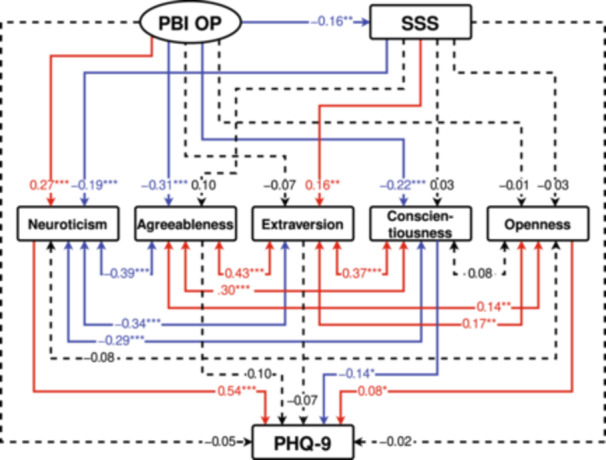
Results of covariance structure analysis in the structural equation model with the Overprotection (OP) subscale on the Parental Bonding Instrument (PBI OP), the subjective social status (SSS) score, and the scores of the five personality traits (neuroticism, agreeableness, extraversion, conscientiousness, and openness) for the NEO Five‐Factor Inventory, as well as depressive symptoms (Patient Health Questionnaire‐9 [PHQ‐9] score) in 404 adult volunteers. Rectangles indicate the observed variables. An oval indicates the latent variable, which consists of two observed variables, namely, paternal and maternal OP subscores (not shown in this figure). Solid arrows indicate statistically significant pathways, red arrows indicate positive effects, and blue arrows indicate negative effects. Dashed lines indicate nonsignificant pathways. The numbers next to the arrows indicate the direct standardized path coefficients (minimum: −1; maximum: +1). *p < 0.05, **p < 0.01, and ***p < 0.001.

Two structural equation models were formulated using the PBI, SSS, and the five factors of the NEO‐FFI, with the total PHQ‐9 score acting as the dependent variable (Figures 1 and 2). In the PBI, the latent variables “Care” and “Overprotection” were created from the two observed variables of “Paternal care” and “Maternal care,” and the two observed variables of “Paternal overprotection” and “Maternal overprotection,” respectively. Furthermore, the total score for the PHQ‐9, the score for the SSS, and the scores for the five personality traits of NEO‐FFI were used as observed variables.

The structural equation modeling was conducted using covariance structure analysis with the robust maximum likelihood estimation method. It introduces latent variables that are difficult to observe directly and can identify associations between latent and observed variables. When conducting covariance structure analysis, as there is no indicator to judge the model's fit, multiple fit indices are combined to make a comprehensive judgement. In this study, the root‐mean‐square error of approximation (RMSEA) and comparative fit index (CFI) were used as fit indices. An “acceptable fit” was defined as an RMSEA of less than 0.08 and a CFI of greater than 0.95, whereas a “good fit” was defined as an RMSEA of less than 0.05 and a CFI of greater than 0.97.^41^ Coefficients in the structural equation models were all standardized (ranging from –1 to +1).

For the comparison of demographic data and questionnaire data, the Student's *t*‐test was conducted or the Pearson correlation coefficient was calculated using SPSS Statistics 28 software. Furthermore, forced‐entry multiple regression analysis was conducted with demographic data (age, sex, education years, current marital status, employment status) and each score for the questionnaire (PBI maternal care, PBI paternal care, PBI maternal overprotection, PBI paternal overprotection, neuroticism, extraversion, openness, agreeableness, conscientiousness, and SSS) as independent variables, and the PHQ‐9 score as the dependent variable.

A *p*‐value of less than 0.05 was considered to indicate a statistically significant difference between groups.

## RESULTS

### Correlation or association between demographic data and each questionnaire score with total PHQ‐9 score

Table 1 presents the results of the analysis of correlations and associations between demographic data and each questionnaire data with total PHQ‐9 score among the 404 adult volunteers. Age negatively correlated with total PHQ‐9 score, and sex and current marital status had significant associations with total PHQ‐9 score. Correlations or associations were not observed between other demographic data and total PHQ‐9 score.

**Table 1:** Characteristics and PHQ‐9, PBI, SSS, and NEO‐FFI scores and their correlation with PHQ‐9 or effects on PHQ‐9 score in 404 adult volunteers.

Characteristic or measure	Number or mean ± SD	Correlation with PHQ‐9 (*r*) or effect on PHQ score (mean ± SD of PHQ‐9, *t*‐test)
Age	42.3 ± 11.9	*r* = –0.139 **
Sex (male : female)	220 : 184	Male 2.9 ± 3.6 vs female 3.7 ± 4.1* (*t*‐test)
Education years	15.2 ± 2.0	*r* = –0.048, n.s. (*t*‐test)
Employment status (employed : unemployed)	341 : 56	Employed 3.3 ± 3.8 vs unemployed 3.6 ± 4.4, n.s. (*t*‐test)
Current marital status (married : unmarried)	287 : 114	Married 3.0 ± 3.7 vs unmarried 4.0 ± 4.1* (*t*‐test)
Living alone (yes : no)	101 : 295	Yes 3.6 ± 4.2 vs no 3.1 ± 3.7, n.s. (*t*‐test)
Comorbidity of physical disease (yes : no)	81 : 319	Yes 3.6 ± 3.7 vs no 3.2 ± 3.9, n.s. (*t*‐test)
PHQ‐9	3.3 ± 3.8	
PBI		
Maternal care	27.7 ± 6.7	*r* = –0.105 *
Paternal care	23.9 ± 7.2	*r* = –0.163 **
Maternal overprotection	10.2 ± 6.8	*r* = 0.095 (n.s.)
Paternal overprotection	9.5 ± 6.1	*r* = 0.081 (n.s.)
NEO‐FFI		
Neuroticism	22.6 ± 7.4	*r* = 0.551**
Extraversion	24.8 ± 6.3	*r* = –0.267**
Openness	27.4 ± 4.5	*r* = 0.032 (n.s.)
Agreeableness	31.5 ± 5.5	*r* = –0.203**
Conscientiousness	28.4 ± 5.1	*r* = –0.300**
SSS	6.1 ± 1.5	*r* = –0.145**

*Note*: Data are presented as means ± SD or numbers. *r* = Pearson correlation coefficient

Abbreviations: NEO‐FFI, NEO Five‐Factor Inventory; PBI, Parental Bonding Instrument; PHQ‐9, Patient Health Questionnaire‐9; SSS, subjective social status (lowest: 1 to highest: 10)

* *p* < 0.05;

** *p* < 0.01; n.s., not significant.

Regarding each PBI subscore, maternal and paternal care were significantly negatively correlated with total PHQ‐9 score. On the other hand, whereas maternal overprotection and paternal overprotection tended to show a positive correlation with total PHQ‐9 score, these correlations were not statistically significant.

Regarding each subscore for the NEO‐FFI, neuroticism was significantly positively correlated with total PHQ‐9 score, and extraversion, agreeableness, and conscientiousness were significantly negatively correlated with total PHQ‐9 score, whereas openness demonstrated no significant correlation with total PHQ‐9 score.

SSS demonstrated a significant negative correlation with total PHQ‐9 score; that is, the higher the SSS, the lower the total PHQ‐9 score.

### Forced‐entry multiple regression analysis with total PHQ‐9 score as a dependent variable

Table 2 demonstrates the results of the forced‐entry multiple regression analysis with total PHQ‐9 score as the dependent variable. The adjusted *R* ^2^ was 0.333. Fifteen independent variables were incorporated into the analysis. Age, neuroticism, agreeableness, and conscientiousness were significantly associated with total PHQ‐9 score, whereas the beta values of the other independent variables were not statistically significant. Multicollinearity was ruled out.

**Table 2:** Results of multiple regression analysis of PHQ‐9 using the forced‐entry method.

Independent factor	Standardized partial regression coefficient (Beta)	*p*‐value	VIF
Age	–0.102	0.036	1.367
Sex	–0.016	0.757	1.551
Education years	–0.024	0.645	1.550
Current marital status	–0.027	0.566	1.261
Employment status	0.049	0.302	1.321
PBI			
Maternal care	–0.036	0.551	2.150
Paternal care	–0.066	0.235	1.761
Maternal overprotection	–0.049	0.457	2.502
Paternal overprotection	–0.045	0.458	2.131
NEO‐FFI			
Neuroticism	0.538	< 0.001	1.565
Extraversion	–0.065	0.209	1.560
Openness	0.071	0.103	1.106
Agreeableness	0.124	0.019	1.589
Conscientiousness	–0.136	0.005	1.344
SSS	–0.020	0.663	1.246
Adjusted *R* ^2^ = 0.333	*F* = 13.879, *p* < 0.001		

*Note*: Dependent factor: PHQ‐9 summary score.

Abbreviations: Beta, standardized partial regression coefficient; NEO‐FFI, NEO Five‐Factor Inventory; PBI, Parental Bonding Instrument; PHQ‐9, Patient Health Questionnaire‐9; SSS, subjective social status; VIF, variance inflation factor.

### Analysis of the structural equation model

In the models, the Care and Overprotection scores for the PBI for each parent were observed variables (not shown in Figures 1 and 2), and “PBI Care” and “PBI Overprotection (OP)” were latent variables, which consist of parental care scores and parental OP scores, respectively (Figures 1 and 2). Moreover, the PHQ‐9, SSS, and the five factors of the NEO‐FFI personality traits were analyzed as observed variables through structural equation models with robust maximum likelihood estimation. Model 1 (Figure 1) had a goodness of fit with an RMSEA of 0.000 and a CFI of 1.000, whereas Model 2 (Figure 2) had a goodness of fit with an RMSEA of 0.028 and a CFI of 0.997. Both models had a good fit.

The standardized coefficients from latent variables to observed variables were 0.631 from “PBI Care” to “Maternal Care,” and 0.701 to “Paternal Care,” 0.760 from “PBI OP” to “Maternal OP,” and 0.759 to “Paternal OP” (data not shown in Figures 1 and 2). In the structural equation models expressing the associations between variables, “PBI Care” exerted a positive direct influence on SSS and a negative direct influence only on neuroticism among the personality traits, and exerted positive direct influences on the other four NEO‐FFI factors (Figure 1). The direct influence of “PBI Care” on PHQ‐9 was not significant. Furthermore, “PBI OP” exerted a negative direct influence on SSS, a positive direct influence on neuroticism, and a negative direct influence on agreeableness and conscientiousness, without having significant direct influences on the other two NEO‐FFI factors (Figure 2). The direct influence of “PBI OP” on PHQ‐9 was not significant. In both models, SSS exerted a negative direct influence on neuroticism and a positive direct influence on extraversion, but did not provide significant direct influences on the other three NEO‐FFI factors (Figures 1 and 2). The direct influence of SSS on PHQ‐9 was not significant (Figures 1 and 2).

**Figure 3:**
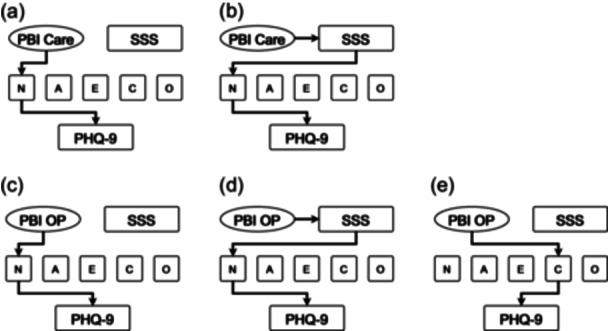
Results of indirect effects, indicating mediation, in the structural equation model with the Parental Bonding Instrument (PBI) Care score, the PBI Overprotection (OP) score, the subjective social status (SSS) score, and the score for the NEO Five‐Factor Inventory personality characteristics (neuroticism [N], agreeableness [A], extraversion [E], conscientiousness [C], and openness [O]), as well as depressive symptoms (Patient Health Questionnaire‐9 [PHQ‐9] score) in 404 adult volunteers. Arrows indicate statistically significant indirect effects. PBI Care, PBI Care factor; PBI OP, PBI Overprotection factor.

Regarding indirect influences, only the statistically significant paths are shown in Figure 3. “PBI Care” demonstrated a significant indirect influence on PHQ‐9 through the combination of SSS and neuroticism (–0.018, *p* = 0.019) (Figure 3b), and the indirect influence through neuroticism alone was also significant (–0.134, *p* < 0.001) (Figure 3a). “PBI OP” demonstrated a significant indirect influence on PHQ‐9 through the combination of SSS and neuroticism (0.016, *p* = 0.036) (Figure 3d). In addition to showing a significant indirect influence on PHQ‐9 through neuroticism alone (0.144, *p* < 0.001) (Figure 3c), “PBI OP” also exerted a significant indirect influence on PHQ‐9 through conscientiousness alone (0.030, *p* = 0.048) (Figure 3e).

In both models, SSS demonstrated a significant negative indirect influence on PHQ‐9 through only neuroticism among the five NEO‐FFI personality traits (–0.097, *p* < 0.001 in Figure 3b; –0.103, *p* < 0.001 in Figure 3d).

Regarding the indirect influences of PBI on the five NEO‐FFI personality traits, “PBI Care” demonstrated a significant negative indirect influence on neuroticism through SSS (–0.034, *p* = 0.016), but did not show significant indirect influences through SSS on the other four NEO‐FFI factors (data not shown). “PBI OP” demonstrated significant indirect influences on neuroticism and extraversion through SSS (0.029, *p* = 0.032; –0.025, *p* = 0.047), but did not demonstrate significant indirect influences on the other three NEO‐FFI factors through SSS (data not shown).

In other words, only neuroticism among the five NEO‐FFI personality traits mediated the influence of SSS on depressive symptoms, whereas the other four traits did not. Furthermore, a significant indirect influence was observed, wherein childhood nurturing experiences impacted neuroticism through SSS and further affected depressive symptoms in adulthood.

It should be noted that *R* ^2^ was almost equivalent in the model of “PBI Care” (0.337) and the model of “PBI OP” (0.338), explaining approximately 34% of the variability in depressive symptoms in adult volunteers.

## DISCUSSION

In this study, we employed structural equation modeling on the data from adult volunteers to investigate the interrelations among childhood nurturing experiences, SSS, personality traits of the FFM (NEO‐FFI), and depressive symptoms. Our results demonstrated that SSS mediates the association between nurturing experiences and the personality trait of neuroticism. Conversely, neuroticism was found to mediate the association between SSS and depressive symptoms, and also between nurturing experiences and depressive symptoms. Furthermore, the complex paths mediated by the two factors of SSS and neuroticism in the association between nurturing experiences and depressive symptoms were identified. No mediation effects of the other four personality traits, excluding the mediation effect of conscientiousness on nurturing experiences and depressive symptoms, were apparent in this model, highlighting the specificity to neuroticism. To our knowledge, this is the first study to date to elucidate such complex indirect effects using FFM personality traits, demonstrating the unique role of neuroticism in the effects of childhood nurturing experiences and SSS on depressive symptoms.

Previous research has indicated personal traits, such as neuroticism, self‐esteem, affective temperament, and resilience, as mediating factors in the association between childhood nurturing experiences and adulthood depressive symptoms.^5^, ^6^, ^12^, ^13^, ^14^ These earlier studies focused on a specific or single personality trait. However, as personality is formed from numerous dimensions,^18^ a comprehensive investigation of the mediating effects without limiting to specific personality traits is essential to understand which traits are associated with this mediation. Therefore, utilizing FFM personality traits, which provide a comprehensive description, instead of a single personality trait, to holistically analyze the mediating effects between childhood nurturing experiences and depressive symptoms is a strength of the present study.

In this study, although we did not find that childhood nurturing experiences affect depressive symptoms by mediation only through SSS, we found that high levels of parental care indirectly reduce depressive symptoms through neuroticism alone or through both paths of SSS and neuroticism. Conversely, high overprotection was found to indirectly enhance depressive symptoms in similar ways. As the mediating effects of SSS and neuroticism were observed for both parental care and overprotection, this highlighted that these mediating effects may be a common phenomenon among the nurturing experiences from parents. Previous research on the association between FFM personality traits and depressive symptoms reported associations with high neuroticism scores, low extraversion scores, and low conscientiousness, whereas agreeableness and openness were not associated with depressive symptoms.^20^, ^21^, ^22^ In the present study, of the FFM personality traits, only neuroticism played a crucial role in the impact of nurturing experiences and SSS on depressive symptoms. Whereas SSS mediated the impact of nurturing experiences on depressive symptoms, this mediating role invariably required the mediating role of neuroticism, but no other personality trait, which is noteworthy. Therefore, when considering the effect of nurturing experienced in childhood on depressive symptoms via SSS, the involvement of neuroticism should always be considered.

High overprotection scores regarding nurturing experienced in childhood also worsened depressive symptoms by reducing conscientiousness. Consistent with the results of this study, lower scores of conscientiousness have been reported in individuals with depression in previous research.^21^ Whereas in our present study we clarified that conscientiousness scores are involved in mediating the association between overprotection in childhood by parents and depressive symptoms, it is noteworthy that parental care during childhood was not associated with this mediating effect. Conscientiousness appears to be affected by subtypes of nurturing, which is in stark contrast to the mediation effect of neuroticism, which does not differ by parental nurturing subtypes.

In our model, the direct effect of SSS on depressive symptoms was not significant; SSS was found to indirectly influence depressive symptoms only through its impact on neuroticism, which is an FFM personality trait. Previous studies have reported self‐esteem and affective temperament as factors mediating the impact of SSS on depressive symptoms.^6^, ^7^ As SSS has been demonstrated to be closely associated with depression and has recently been attracting attention from the viewpoint of etiology,^6^, ^7^, ^28^, ^29^ it is imperative to elucidate which personality traits are associated with SSS, or which personality traits mediate the effect of SSS on depression. However, the crucial question of which of the numerous personality traits mediate the impact of SSS on depression, and which do not, had not been addressed previously. In this study, we comprehensively evaluated various personality traits, included all five traits of FFM personality applicable to general adults in the analysis, and demonstrated for the first time, to our knowledge, that only one factor, namely, neuroticism, is a significant mediator in the association between SSS and depressive symptoms, whereas the other four factors had no significant impact. Moreover, supporting part of the results of this study, it has been reported that high neuroticism is associated with low SSS and low income.^42^ Therefore, the mediating effects of self‐esteem and affective temperament, which have been demonstrated in previous studies, suggest that these personality traits have features common to neuroticism, and indeed, our data demonstrated that neuroticism correlates most strongly with self‐esteem and affective temperament among the FFM (data not shown). The results of our study are expected to provide a starting point for elucidating the mechanisms by which SSS affects depression. In the future, it will be necessary to perform studies from the perspective of the mediating effects of neuroticism, whose association with depression was clearly demonstrated,^4^ to clarify the association of SSS with depression or depressive symptoms.

The results of the present study indicate the potential usefulness of understanding an individual's nurturing experiences in childhood, SSS, and neuroticism to predict and prevent the onset of depression. Whereas it is impossible to alter the childhood nurturing experiences of individuals in adulthood, it is possible to increase their SSS and lower their neuroticism. Improvements in objective socioeconomic status, such as education and income, which are associated with SSS, have been suggested to enhance SSS.^32^, ^43^ Additionally, whereas psychological interventions that increase SSS have not yet been developed for clinical implementation, there are psychological manipulations that affect SSS,^44^ and they may be clinically implemented in the future. For people with high neuroticism scores, cognitive behavioral therapy to enhance emotional regulation skills may be effective. Psychological treatments based on mindfulness, such as mindfulness‐based cognitive therapy, have demonstrated significant neuroticism‐reducing effects.^45^, ^46^ Another effective intervention is pharmacological, that is, serotonergic drugs can reduce neuroticism.^47^ Furthermore, increasing physical activity and reducing workplace harassment, among other environmental adjustments, have been reported to decrease neuroticism.^48^, ^49^ Considering the structural equation model of this study, it is presumed that the various interventions mentioned above may contribute in a chain reaction towards the improvement of depressive symptoms in adults, which warrants verification in future clinical trials.

## LIMITATIONS

The retrospective evaluation of childhood experiences in this study may be influenced by recall bias, as it relies on the subjects' memories. In addition, as this study used a cross‐sectional design, causal associations cannot be concluded. Therefore, it is necessary to confirm whether similar results can be obtained through prospective studies that objectively evaluate childhood nurturing experiences, and follow children longitudinally. Moreover, as the sample mainly comprises healthy adults and not patients with depression, there may be limitations in applying the model of this study to patients with depression.

## CONCLUSION

In this study, we suggested through a structural equation model that the impact of the nurturing by parents experienced during childhood on neuroticism in adulthood is mediated by SSS, leading to varying levels of depressive symptoms in adulthood through their impacts on neuroticism. Although it has recently been demonstrated that SSS affects psychiatric disorders and symptoms, our study suggests that the underlying mechanism is partly through the influence of SSS on neuroticism. Elucidating the mechanism of the association between SSS and psychiatric disorders in the future is expected to be beneficial towards clarifying the mechanisms of psychiatric disorders and for developing treatments.

## AUTHOR CONTRIBUTIONS

**Yuki Nakagawa**: Conceptualization; data curation; validation; formal analysis; investigation; methodology; project administration; visualization; writing—original draft; writing—review and editing. **Miki Ono**: Data curation; writing—original draft; writing—review and editing. **Chihiro Morishita**: Data curation; writing—original draft; writing—review and editing. **Mina Honyashiki**: Data curation; writing—original draft; writing—review and editing. **Yu Tamada**: Data curation; writing—original draft; writing—review and editing. **Yota Fujimura**: Data curation; writing—original draft; writing—review and editing. **Shinji Higashi**: Data curation; writing—original draft; writing—review and editing. **Naoki Hashimoto**: Data curation; writing—original draft; writing—review and editing. **Takeshi Inoue**: Conceptualization; data curation; validation; formal analysis; investigation; methodology; project administration; writing—original draft; writing—review and editing. **Jiro Masuya**: Data curation; writing—original draft; writing—review and editing.

## CONFLICT OF INTEREST STATEMENT

The authors have read the journal's policy and the authors of this manuscript have the following competing interests: Yu Tamada has received personal fees from Otsuka Pharmaceutical, Sumitomo Pharma, Eisai, MSD, and Meiji Seika Pharma. Yota Fujimura has received personal compensation from Sumitomo Pharma, and grants from Otsuka Pharmaceutical, Sumitomo Pharma, and Shionogi. Shinji Higashi has received personal fees from Dainippon Sumitomo Pharma, Eisai, Takeda Pharmaceutical, Shionogi, Viatris, and MSD. Naoki Hashimoto has received personal fees from Janssen Pharmaceutical, Yoshitomiyakuhin, Otsuka Pharmaceutical, Sumitomo Pharma, Takeda Pharmaceutical, and Meiji Seika Pharma. Takeshi Inoue has received personal fees from Mochida Pharmaceutical, Takeda Pharmaceutical, Eli Lilly, Janssen Pharmaceutical, MSD, Taisho Toyama Pharmaceutical, Yoshitomiyakuhin, and Daiichi Sankyo; grants from Shionogi, Astellas, Tsumura, and Eisai; and grants and personal compensation from Otsuka Pharmaceutical, Sumitomo Pharma, Mitsubishi Tanabe Pharma, Kyowa Pharmaceutical Industry, Pfizer, Novartis Pharma, and Meiji Seika Pharma; and is a member of the advisory boards of Pfizer, Novartis Pharma, and Mitsubishi Tanabe Pharma. Jiro Masuya has received personal fees from Otsuka Pharmaceutical, Eli Lilly, Astellas, and Meiji Yasuda Mental Health Foundation, and grants from Pfizer. The remaining authors declare no conflict of interest.

## ETHICS APPROVAL STATEMENT

The study protocol was approved by the Ethics Committee of Tokyo Medical University (SH3308) and Hokkaido University Hospital (013‐0184).

## PATIENT CONSENT STATEMENT

All subjects were informed that participation in this research was voluntary and the collected information was anonymized so that the individuals could not be identified. Only the subjects who gave their consent to participate in this study were analyzed.

## CLINICAL TRIAL REGISTRATION

N/A.

## Data Availability

The data used in this study cannot be shared publicly because of Ethics Committee restriction. All relevant data are within the paper. Data are available from the Internal Review Board of the Department of Psychiatry, Tokyo Medical University, Japan (contact via email: seisinka@tokyo-med.ac.jp), for researchers who meet the criteria for access to confidential data.

## References

[1] Ishikawa H , Tachimori H , Takeshima T , Umeda M , Miyamoto K , Shimoda H , et al. Prevalence, treatment, and the correlates of common mental disorders in the mid 2010's in Japan: the results of the world mental health Japan 2nd survey. J Affect Disord. 2018;241:554–562. 30153639 10.1016/j.jad.2018.08.050

[2] Caspi A , Sugden K , Moffitt TE , Taylor A , Craig IW , Harrington H , et al. Influence of life stress on depression: moderation by a polymorphism in the 5‐HTT gene. Science. 2003;301:386–389. 12869766 10.1126/science.1083968

[3] Alloy LB , Abramson LY , Walshaw PD , Keyser J , Gerstein RK . A cognitive vulnerability‐stress perspective on bipolar spectrum disorders in a normative adolescent brain, cognitive, and emotional development context. Dev Psychopathol. 2006;18:1055–1103. 17064429 10.1017/S0954579406060524

[4] Kendler KS , Kuhn J , Prescott CA . The interrelationship of neuroticism, sex, and stressful life events in the prediction of episodes of major depression. Am J Psychiatry. 2004;161:631–636. 15056508 10.1176/appi.ajp.161.4.631

[5] Kendler KS , Gardner CO . Sex differences in the pathways to major depression: a study of opposite‐sex twin pairs. Am J Psychiatry. 2014;171:426–435. 24525762 10.1176/appi.ajp.2013.13101375 PMC3972260

[6] Hayashida T , Higashiyama M , Sakuta K , Masuya J , Ichiki M , Kusumi I , et al. Subjective social status via mediation of childhood parenting is associated with adulthood depression in non‐clinical adult volunteers. Psychiatry Res. 2019;274:352–357. 30851598 10.1016/j.psychres.2019.02.061

[7] Higashiyama M , Hayashida T , Sakuta K , Fujimura Y , Masuya J , Ichiki M , et al Complex effects of childhood abuse, affective temperament, and subjective social status on depressive symptoms of adult volunteers from the community. Neuropsychiatr Dis Treat. 2019;15:2477–2485. 31695384 10.2147/NDT.S209100 PMC6717723

[8] Parker G , Hadzi‐Pavlovic D , Greenwald S , Weissman M . Low parental care as a risk factor to lifetime depression in a community sample. J Affect Disord. 1995;33:173–180. 7790669 10.1016/0165-0327(94)00086-o

[9] Finzi‐Dottan R , Karu T . From emotional abuse in childhood to psychopathology in adulthood: a path mediated by immature defense mechanisms and self‐esteem. J Nerv Ment Dis. 2006;194:616–621. 16909071 10.1097/01.nmd.0000230654.49933.23

[10] Weich S , Patterson J , Shaw R , Stewart‐Brown S . Family relationships in childhood and common psychiatric disorders in later life: systematic review of prospective studies. Br J Psychiatry. 2009;194:392–398. 19407266 10.1192/bjp.bp.107.042515

[11] Hayashi Y , Okamoto Y , Takagaki K , Okada G , Toki S , Inoue T , et al. Direct and indirect influences of childhood abuse on depression symptoms in patients with major depressive disorder. BMC Psychiatry. 2015;15:244. 26467656 10.1186/s12888-015-0636-1 PMC4604614

[12] Ono Y , Takaesu Y , Nakai Y , Ichiki M , Masuya J , Kusumi I , et al. The influence of parental care and overprotection, neuroticism and adult stressful life events on depressive symptoms in the general adult population. J Affect Disord. 2017;217:66–72. 28391110 10.1016/j.jad.2017.03.058

[13] Toyoshima K , Inoue T , Masuya J , Fujimura Y , Higashi S , Kusumi I . Associations among childhood parenting, affective temperaments, depressive symptoms, and cognitive complaints in adult community volunteers. J Affect Disord. 2020;276:361–368. 32871666 10.1016/j.jad.2020.07.107

[14] Ito S , Morishita C , Masuya J , Ono M , Honyashiki M , Higashi S , et al. Moderating and mediating effects of resilience together with neuroticism on depressive symptoms in adult volunteers. Neuropsychiatr Dis Treat. 2022;18:1751–1761. 36000024 10.2147/NDT.S370201 PMC9393030

[15] Digman JM . Personality structure: emergence of the five‐factor model. Annu Rev Psychol. 1990;41:417–440.

[16] Goldberg LR . The structure of phenotypic personality traits. Am Psychol. 1993;48:26–34. 8427480 10.1037//0003-066x.48.1.26

[17] Markon KE , Krueger RF , Watson D . Delineating the structure of normal and abnormal personality: an integrative hierarchical approach. J Pers Soc Psychol. 2005;88:139–157. 15631580 10.1037/0022-3514.88.1.139 PMC2242353

[18] Costa PT , McCrae RR . The NEO‐PI/NEO‐FFI Manual Supplement. Odessa: Psychological Assessment Resources; 1989.

[19] Shimonaka J , Nakazato K , Gondo Y , Takayama M . NEO‐PI‐R, NEO‐FFI Manual for the Japanese Version Revised and enlarged edition. Tokyo Shinri,Inc.; 2011.

[20] Clark LA , Watson D . Temperament: a new paradigm for trait psychology. In: Pervin L , John O eds Handbook of Personality. 2nd ed. Guilford; 1999. p. 399–423.

[21] Kotov R , Gamez W , Schmidt F , Watson D . Linking “big” personality traits to anxiety, depressive, and substance use disorders: a meta‐analysis. Psychol Bull. 2010;136:768–821. 20804236 10.1037/a0020327

[22] Klein DN , Kotov R , Bufferd SJ . Personality and depression: explanatory models and review of the evidence. Annu Rev Clin Psychol. 2011;7:269–295. 21166535 10.1146/annurev-clinpsy-032210-104540 PMC3518491

[23] Kanbayashi H . Subjective social status and health: a review from a perspective of status identification study. J Hum Hypertens. 2016;21:59–82.

[24] Adler NE , Epel ES , Castellazzo G , Ickovics JR . Relationship of subjective and objective social status with psychological and physiological functioning: preliminary data in healthy white women. Health Psychol. 2000;19:586–592. 11129362 10.1037//0278-6133.19.6.586

[25] Ostrove JM , Adler NE , Kuppermann M , Washington AE . Objective and subjective assessments of socioeconomic status and their relationship to self‐rated health in an ethnically diverse sample of pregnant women. Health Psychol. 2000;19:613–618. 11129365 10.1037//0278-6133.19.6.613

[26] Singh‐Manoux A , Adler NE , Marmot MG . Subjective social status: its determinants and its association with measures of ill‐health in the Whitehall II study. Soc Sci Med. 2003;56:1321–1333. 12600368 10.1016/s0277-9536(02)00131-4

[27] Operario D , Adler NE , Williams DR . Subjective social status: reliability and predictive utility for global health. Psychol Health. 2004;19:237–246.

[28] Scott KM , Al‐Hamzawi AO , Andrade LH , Borges G , Caldas‐de‐Almeida JM , Fiestas F , et al. Associations between subjective social status and DSM‐IV mental disorders: results from the World Mental Health surveys. JAMA Psychiatry. 2014;71:1400–1408. 25354080 10.1001/jamapsychiatry.2014.1337 PMC5315238

[29] Hoebel J , Maske UE , Zeeb H , Lampert T . Social inequalities and depressive symptoms in adults: the role of objective and subjective socioeconomic status. PLoS One. 2017;12:e0169764. 28107456 10.1371/journal.pone.0169764 PMC5249164

[30] Cundiff JM , Smith TW , Uchino BN , Berg CA . Subjective social status: construct validity and associations with psychosocial vulnerability and self‐rated health. Int J Behav Med. 2013;20:148–158. 22200973 10.1007/s12529-011-9206-1

[31] Parker G , Tupling H , Brown LB . A parental bonding instrument. Br J Med Psychol. 1979;52:1–10.

[32] Goodman E , Maxwell S , Malspeis S , Adler N . Developmental trajectories of subjective social status. Pediatrics. 2015;136:e633–e640. 26324868 10.1542/peds.2015-1300 PMC4552092

[33] Spitzer RL , Kroenke K , Williams JB . The Patient Health Questionnaire Primary Care Study Group. Validation and utility of a self‐report version of PRIME‐MD: the PHQ primary care study. JAMA. 1999;282:1737–1744. 10568646 10.1001/jama.282.18.1737

[34] Muramatsu K , Kamijima K , Yoshida M , Otsubo T , Miyaoka H , Muramatsu Y , et al. The patient health questionnaire, Japanese version: validity according to the mini‐international neuropsychiatric interview‐plus. Psychol Rep. 2007;101:952–960. 18232454 10.2466/pr0.101.3.952-960

[35] Wilhelm K , Niven H , Parker G , Hadzi‐Pavlovic D . The stability of the parental bonding instrument over a 20‐year period. Psychol Med. 2005;35:387–393. 15841874 10.1017/s0033291704003538

[36] Kitamura T , Suzuki T . A validation study of the parental bonding instrument in a Japanese population. Psychiatry Clin Neurosci. 1993;47:29–36. 10.1111/j.1440-1819.1993.tb02026.x 8411788

[37] Costa PT Jr , McCrae RR . Revised NEO Personality Inventory (NEO‐PI‐R) and NEO Five‐Factor Inventory (NEO‐FFI) professional manual. Psychological Assessment Resources; 1992.

[38] Shimonaka J , Nakazato K , Gondo Y , Takayama M . Japanese version of NEO‐PI‐R and short version of NEO‐FFI. In: Tsujisai S ed Theory and practice of 5 factor personality test. Kitaoji Shoten; 1998. p. 47–59.

[39] Yoshimura K , Ono Y , Nakamura K , Nathan JH , Suzuki K . Validation of the Japanese version of the NEO five‐factor inventory in a large community sample. Psychol Rep. 2001;88:443–449. 11351886 10.2466/pr0.2001.88.2.443

[40] Tsuno K , Kawakami N , Tsutsumi A , Shimazu A , Inoue A , Odagiri Y , et al. Socioeconomic determinants of bullying in the workplace: a national representative sample in Japan. PLoS One. 2015;10:e0119435. 25751252 10.1371/journal.pone.0119435 PMC4353706

[41] Schermelleh‐Engel K , Moosbrugger H , Müller H . Evaluating the fit of structural equation models: tests of significance and descriptive goodness‐of‐fit measures. MPR Online. 2003;8:23–74.

[42] Alfonsi G , Conway M , Pushkar D . The lower subjective social status of neurotic individuals: multiple pathways through occupational prestige, income, and illness. J Pers. 2011;79:619–642. 21534966 10.1111/j.1467-6494.2011.00684.x

[43] Zvolensky MJ , Paulus DJ , Bakhshaie J , Garza M , Manning K , Lemaire C , et al. Anxiety sensitivity and age: roles in understanding subjective social status among low income adult latinos in primary care. J Immigr Minor Health. 2018;20:632–640. 28681307 10.1007/s10903-017-0623-3 PMC11846059

[44] Cheon BK , Hong YY . Mere experience of low subjective socioeconomic status stimulates appetite and food intake. Proc Natl Acad Sci U S A. 2017;114:72–77. 27994148 10.1073/pnas.1607330114 PMC5224403

[45] Sauer‐Zavala S , Wilner JG , Barlow DH . Addressing neuroticism in psychological treatment. Personal Disord. 2017;8:191–198. 29120218 10.1037/per0000224

[46] Armstrong L , Rimes KA . Mindfulness‐based cognitive therapy for neuroticism (stress vulnerability): a pilot randomized study. Behav Ther. 2016;47:287–298. 27157024 10.1016/j.beth.2015.12.005

[47] Quilty LC , Meusel LAC , Bagby RM . Neuroticism as a mediator of treatment response to SSRIs in major depressive disorder. J Affect Disord. 2008;111:67–73. 18384882 10.1016/j.jad.2008.02.006

[48] Nakajima K , Shimura A , Kikkawa M , Ito S , Honyashiki M , Tamada Y , et al. Optimal physical activity is associated with the reduction of depressive symptoms via neuroticism and resilience. Healthcare. 2023;11:1900. 37444734 10.3390/healthcare11131900 PMC10340455

[49] Persson R , Høgh A , Grynderup MB , Willert MV , Gullander M , Hansen ÅM , et al. Relationship between changes in workplace bullying status and the reporting of personality characteristics. J Occup Environ Med. 2016;58:902–910. 27454394 10.1097/JOM.0000000000000822

